# Pediatric Metabolic Syndrome: From Prevention to Treatment

**DOI:** 10.1155/2012/374168

**Published:** 2012-11-11

**Authors:** Roya Kelishadi, Parinaz Poursafa, Sarah D. de Ferranti, Peter Schwandt, Khosrow Adeli, Altan Onat, Samuel S. Gidding

**Affiliations:** ^1^Pediatrics Department, Child Growth and Development Research Center, Isfahan University of Medical Sciences, 81676-36954 Isfahan, Iran; ^2^Environmental Engineering Department, Environment Research Center, Isfahan University of Medical Sciences, Isfahan, Iran; ^3^Preventive Cardiology Department, Children's Hospital Boston, Boston, MA, USA; ^4^Arteriosklerose-Präventions-Institut, University of Munich, Munich, Germany; ^5^Clinical Biochemistry, The Hospital for Sick Children, University of Toronto, Toronto, ON, Canada; ^6^Cardiology Department, Cerrahpasa Medical Faculty, Istanbul University, Istanbul, Turkey; ^7^Nemours Cardiac Center, Alfred I. DuPont Children's Hospital and Thomas Jefferson University, Wilmington, DE, USA

Pediatric metabolic syndrome is becoming a substantial health problem at global level [1, 2]. It has a complex multifactorial etiology. Prevention and control of its modifiable risk factors from prenatal period can have long-term health effect on primordial prevention of chronic noncommunicable diseases. Given the increasing evidence on tracking of risk factors from childhood into adult life, the potential role of genetic, prenatal, environmental, biological, and behavioral determinants of pediatric metabolic syndrome should be underscored [3–5]. 

Pediatric metabolic syndrome is mainly related to “globesity,” a term used by the World Health Organization to focus on the escalating global epidemic of overweight and obesity [6]. Although most cases are secondary to obesity, actually a substantial number of normal-weight children and adolescents have at least some components of this syndrome [7]. The environmental factors, gene-gene, and gene-environment interactions should be considered in this context. 

 A growing body of evidence proposes that nonalcoholic fatty liver disease (NAFLD) and pediatric metabolic syndrome are interrelated and have common pathophysiological features [8–10]. The “two-hit hypothesis” is the most widely accepted model explaining the progression of NAFLD [11], and may also have a role in the development of the metabolic syndrome. Oxidative stress and proinflammatory cytokines are of the main factors initiating the second hit; the association of environmental influences on these factors, even in the pediatric age group [12–14].

 The other aspect of the influences of environmental factors on the development of pediatric metabolic syndrome can be the impact of these factors, as air pollutants, on intrauterine growth retardation, low birth weight, and prematurity [15, 16], and the impact of other factors as noise pollution and passive smoking on components of pediatric metabolic syndrome [17], which in turn can be associated with higher risk of chronic diseases in later life. Furthermore, currently many environmental obesogens are identified; they are classified as chemical simulators of metabolic hormones or brain neurotransmitters [18, 19]. All these mechanisms propose that the systemic responses to long-term exposure to environmental factors could potentially increase the risk for development of the pediatric metabolic syndrome. 

 Interventions including community involvement can be useful in improving health at individual and public health levels [20]. Prevention and control of modifiable risk factors as air and noise pollution, passive smoking, overweight, and unhealthy lifestyle, along with primordial prevention by good pregnancy care for prevention of low birth weight, encouraging breast feeding, and using healthy complimentary foods during infancy can impact the overall health of children and adolescents as well as the prevention and control of pediatric metabolic syndrome and its treatment modalities. 

This special issue is dedicated to increasing the depth of research across all areas of the pediatric metabolic syndrome, and highlights the preventive measures as well as management by nonpharmacological and pharmacological treatment.

*Roya Kelishadi* *Parinaz Poursafa* *Sarah D. de Ferranti* *Peter Schwandt* *Khosrow Adeli* *Altan Onat* *Samuel S. Gidding*
